# The HIV-1 Gag Protein Displays Extensive Functional and Structural Roles in Virus Replication and Infectivity

**DOI:** 10.3390/ijms23147569

**Published:** 2022-07-08

**Authors:** Veronna Marie, Michelle Lucille Gordon

**Affiliations:** KwaZulu-Natal Research, Innovation and Sequencing Platform, University of KwaZulu-Natal, Durban 4041, South Africa; tarinm@ukzn.ac.za

**Keywords:** Gag molecular structure, p17 Gag, p24 Gag, p7 Gag, p6 Gag, p2 and p1 Gag

## Abstract

Once merely thought of as the protein responsible for the overall physical nature of the human immunodeficiency virus type 1 (HIV-1), the Gag polyprotein has since been elucidated to have several roles in viral replication and functionality. Over the years, extensive research into the polyproteins’ structure has revealed that Gag can mediate its own trafficking to the plasma membrane, it can interact with several host factors and can even aid in viral genome packaging. Not surprisingly, Gag has also been associated with HIV-1 drug resistance and even treatment failure. Therefore, this review provides an extensive overview of the structural and functional roles of the HIV-1 Gag domains in virion integrity, functionality and infectivity.

## 1. Introduction

**Figure 1:**
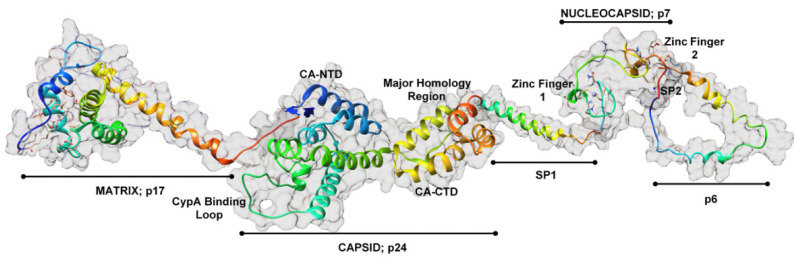
Schematic representation of a rod-shaped HIV-1 Gag polyprotein obtained by the concatenation of previously resolved structures of individual Gag domains. PDB IDs: matrix (2h3i; [12]); capsid (3nte; [13]); SP1 (1u57; [14]); nucleocapsid (1a1t; [15]); p6 (2c55; [16]). Note: The matrix-capsid linker and SP2 are hypothetical representations.

In the human immunodeficiency virus type 1 (HIV-1), the autocatalytic cleavage of the aspartyl protease enzyme from Gag–Pol (PR160) prompts the cleavage of both Gag-Pol and Gag (PR55) at predefined sites in the polyprotein [1]. Group specific antigen, commonly referred to as Gag, is the HIV-1 polyprotein associated with viral structure and infectivity [2]. The 500 amino acid protein comprises four main structural domains (matrix, capsid, nucleocapsid, p6) and two small spacer peptides (SP1 and SP2) that aid in virion formation [2] as shown theoretically in Figure 1 below. For successful maturation to occur, a systematic proteolytic cleavage of Gag–Pol and Gag [3] is imperative. Furthermore, RNA dimerization as well as accurate RNA–Gag interactions are vital for the proteolytic cleavage of the substrate and enzyme [4].

Upon translation from the 9 kb transcript in the cytoplasm [5], Gag is trafficked to the plasma membrane where the membrane-bound polyprotein recruits and packages two copies of viral RNA per virion [6]. At the site of assembly, Gag binds cell membranes, undergoes multimerization and finally buds. Host factors employed by Gag facilitate scission and release of the immature viral particles from the cell membrane [6]. As a result, Gag and thereby its domains have several functional roles in viral replication and infectivity as summarized in Table 1.

Structural studies focusing on Gag and its assembly have furthered our understanding of Gag domain interactions with the host as well as with each other [4]. In particular, studies on individual Gag domains such as matrix, capsid and nucleocapsid in both mature and immature virions have been evaluated [7]. While it has been postulated that amino acid variation is observed in HIV-1 non-B vs. B subtypes [8], sequence analysis and structural studies indicate that strategic amino acid substitutions in Gag may also contribute to drug resistance [9,10,11].

Therefore, this paper will review the various structural and functional roles of the Gag domains in the viral maturation process, and thereby infectivity.

## 2. Matrix

The matrix (MA; p17) protein is a 132 amino acid polypeptide that lines the inner surface of the virion lipid bilayer [4]. Structurally, MA consists of an amino-terminal (N-terminal) globular head and a flexible carboxyl-terminal (C-terminal) tail [17,18]. In MA, the globular head is composed of a 3_10_ helix and four α-helices that are capped by a three-stranded β-sheet. A fifth α-helix connects the capsid and MA domains which project away from the β-sheet, exposing the C-terminal residues and forming the tail [19]. Based on the ionic strength and concentration of protein buffers, MA may exist as either monomers, dimers and trimers or oligomers of monomers, dimers and trimers [20].

### 2.1. MA and Myristoylation

**Figure 2:**
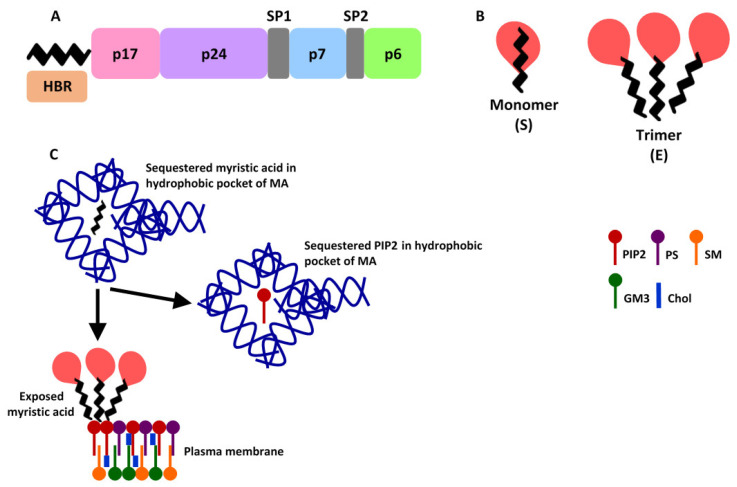
Proposed HIV-1 myristoylation at the molecular level. (**A**) Full length Gag polyprotein with a myristoylated MA at the N-terminal domain. (**B**) Sequestered (S) and Exposed (E) transition states in different MA conformations [37]. (**C**) The sequestered myristic acid switches to exposed upon PIP_2_ binding at the plasma membrane. The PIP_2_ fatty acid is then sequestered into the vacant hydrophobic pocket (adapted from [38]).

Functionally, the flexible nature of MA [21] allows for the targeting of the precursor Gag–Pol and the precursor PR55 Gag to the plasma membrane [22]. This process requires two important chemical signals, namely (i) myristoylation and (ii) the plasma membrane-specific phosphatidylinositol-4,5-bisphosphate (PIP_2_) [6]. Firstly, myristoylation occurs when *N-methyltransferase* catalyses a co-translational covalent bond between myristic acid and the G2 amino acid residue of the N-terminal domain (within the MGXXX{S/T}XX consensus sequence) of MA (Figure 2A) [4]. Since myristoylation increases the affinity of MA to the plasma membrane, it is not only critical for membrane targeting but for the production of immature, non-infectious virions [23]. Disruption of the myristoylation signal by mutations, such as G2A, can affect membrane targeting and virion production [24]. During myristoylation, myristic acid undergoes two reversible conformational transitions, known as the “myristoylation switch”. These transitions are classified as exposed or sequestered within a hydrophobic pocket (Figure 2B) [25]. The hydrophobic pocket comprising the sequestered myristic acid is formed by the α-helices of MA [26]. As the myristoylation switch is dependent on several amino acid residues, particularly V7 and L8 [27], mutations at these positions can affect membrane targeting of Gag as well as inhibition of viral assembly and release [28]. In addition to these, the capsid domain, pH and the rate of calmodulin-binding may also impact the exposure of myristic acid [29].

The second signal involving PIP_2_ and a cluster of basic residues localised around the cationic loop connecting β-strands one and two, otherwise known as the highly basic region (HBR; amino acids 17–31) [30], stabilizes membrane binding to the inner leaflet of the plasma membrane [31]. The HBR facilitates an electrostatic interaction between the protein and the negatively charged PIP_2_ in the plasma membrane as seen in Figure 2C [30]. The exposure of myristic acid, and thereby its insertion into the lipid bilayer, is influenced by the HBR and the PIP2 head group [25]. Upon transition from sequestered to exposed, the PIP_2_ ^30^KLKH^34^ recognition site sequesters the PIP_2_ fatty acid into the hydrophobic pocket (Figure 2C). The interaction between the plasma membrane and myristic acid thereby promotes the formation of a bi-directional lipid bilayer [4].

Although myristic acid exposure has been almost exclusively associated with MA trimerization (Figure 2B) [32,33], Valentine et al. [34] showed that after encapsidation into reverse micelles, myristic acid exposure could occur in monomer forms. Interestingly, molecular dynamic simulations conducted by Monje-Galvan and Voth [26] showed that while MA monomers did not form trimers, monomer interactions increased the concentration of PIP_2_ and recruited it to the binding site, thereby enhancing viral assembly. Apart from the interactions between myristic acid and PIP_2_, un-cleaved PR55 Gag multimerization may enhance its membrane binding activity through MA, indicating complex interactions between MA, capsid and the nucleocapsid domains (extensively reviewed in [35]). Furthermore, Wen et al. [36] showed that PR55 Gag can selectively target pre-enriched PIP_2_ membranes for viral assembly of the immature viral particles, while the Gag multimerization process further promoted PIP_2_ clustering. However, in spite of these revelations, it is clear that the role of MA in membrane targeting is a complex process that is affected by numerous factors.

### 2.2. Other MA Functions

During the early stages of viral replication, MA is also associated with reverse transcription as well as the pre-integration complex (PIC) [39]. The PIC is a large nucleoprotein composed of several viral and cellular (host) proteins that allow integration during replication [40]. In addition to viral heterologous DNA and ribosomal RNA (rRNA), MA also binds to host transfer RNAs (tRNAs) [41]. A study conducted by Bou-Nadar et al. [41] demonstrated that aromatic and basic residues present within MA are preconfigured to recognize tRNA elbows. Though the authors indicate that it is highly improbable that tRNA and membrane binding occurs simultaneously, point substitutions that abolished MA–tRNA binding resulted in large scale redistribution of Gag to the plasma membrane and reduced HIV-1 replication [41]. Interestingly, MA also has cytokine-like functions that act on pre-activated human T cells encouraging proliferation, viral replication after p17R host-cellular receptor binding and pro-inflammatory cytokine release [42,43]. Therefore, MA may have several important known and/or unknown roles in the virus- and host-derived factors that contribute to a favourable HIV-1 infection and replication cycle [44]. Furthermore, it has been suggested that Env incorporation into the budding viral particles is mediated through interactions between the precursor PR55 Gag MA and the cytoplasmic tail of Env (gp41) [6]. This was substantiated by point mutations and deletions in MA that adversely affected Env incorporation without influencing particle formation [45,46]. In addition, biochemical analyses conducted by Tedbury et al. [47] showed that Env incorporation was negatively impacted by MA’s inability to form trimers, thus indicating a correlation between MA trimerization and Env incorporation. However, the direct mechanism on Env incorporation remains unclear [6].

## 3. Capsid

In a mature virion, the HIV-1 capsid (CA, p24) protein oligomerizes to form a shell around the virus’s RNA and core-associated proteins [48]. All morphological variances of the CA domain are derived from a collection of hexameric (rings) CA monomers [49] that have the same tertiary structure [50]. CA comprises two separate domains: the N-terminal domain (CA_NTD 1–145_) and the C-terminal domain (CA_CTD 151–231_) [4], connected by a flexible inter-domain linker (residues 146–150) [51,52]. Structural studies revealed that CA is predominantly composed of α-helices, with seven and four α-helices in CA_NTD_ and CA_CTD_, respectively [53]. Moreover, the NTD of CA contains an amino-terminal β-hairpin and resembles an arrowhead, whereas the globular CTD comprises one 3_10_ helix [53]. Extensive intrahexameric NTD–NTD and NTD–CTD interactions stabilize the central CA rings [53]. Accordingly, the first three helices of CA comprising the NTD–NTD interacting residues form a loose 18-helix central barrel that encompasses a hydrophobic core of aliphatic sidechains. In addition to a hydrophobic component, NTD–CTD interactions are primarily formed by sidechains extending from helix 8 of the CTD to helix 4 of the NTD. Further contacts are also made between helix 7 (C-terminal end) and helix 11 of the CTD [54]. Flexible CTD–CTD interactions link the adjacent hexameric rings, thus allowing for the formation of the curved lattice [55]. Additionally, it has also been shown that the inositol hexaphosphate (IP6) metabolite has a significant role in stabilizing the CA hexamers upon incorporation [56]. Recently, Renner et al. [57] showed that the coordination of IP6 to promote the assembly of mature CAs capable of reverse transcription and infection was mediated by both arginine and lysine ring binding. Furthermore, the asymmetrical structure infers that no two CAs are identical in capsid [58]. In addition to hexamers, formation of the mature “fullerene cone” of capsids is also dependent on the incorporation of 12 CA protein pentamers. Zhao et al. [58] showed that pentamer incorporation results in proximal trimer contacts by way of sharp bite angles (approximately 137° between pentamer–hexamer subunits), thereby inducing acute surface curvature. Noteworthy, Mattei et al. [59] suggest that either the angle of curvature determines pentamer position or the pentamer positions are determined by the angle of curvature, as pentamers were present at sites of great curvature. Therefore, the flexibility and complex molecular interactions of CA support a variety of conformations that ultimately contribute to structural variation within the protein itself [60].

### 3.1. CA, Cyclophilin A and Other Host Factors

The flexible nature of CA also allows for the interaction of a range of host factors such as CPSF6, NUP153, NUP358, TRIM5α and MxB [61]. One of the more notable host factors that binds to the CA domain of the precursor PR55 Gag is the proline isomerase cyclophilin A (CypA) [62]. An extended proline-rich loop between the fourth and fifth helices in the NTD compose the binding site for CypA [63]. The CypA–CA stoichiometry is approximately 1:10 per particle [64] with residue P90 being the most important amino acid for CypA binding [65]. While the role of CypA is still debatable [66], it has been suggested that the addition of CypA into CA may be an important defence mechanism against antiviral innate immune responses after viral entry into the host cells [67]. A structural study conducted by Liu et al. [68] showed that CypA is not only recruited to facilitate HIV-1 infection but to also stabilize CA itself. Interestingly, although CypA is imperative for group M HIV-1 replication, group O variants are insensitive to CypA-inhibiting drugs and replicate efficiently in CypA-deficient cells [63]. Consequently, the role of CypA in specific group M HIV-1 replication versus its absence in other groups is still largely unknown.

Since reverse transcription is carried out inside the mature CA, dynamic, charged pores positioned at the centre of each CA multimer allows for the import of nucleotides [57]. In addition to IP6, Xie et al. [69] demonstrated that CA interacts with cellular nucleoporins, including NUP358, to allow import of the PIC into the nucleus. Additionally, it has been shown that partially(intact) viral cores enter the nucleus by directly interacting with CPSF6, the disruption of which results in altered target sites for HIV-1 integration [70].

### 3.2. CA and the Major Homology Region

Each CA domain has a different function in HIV-1 morphogenesis. Although the NTD of CA is not necessary for immature virion assembly, it is indispensable for mature core formation [71]. Contrastingly, the CTD of CA is important for both the formation of the core in mature particles and in virion assembly in immature particles [72]. In all retroviruses, a conserved sequence known as the major homology region (MHR) is located in the CTD of the precursor PR55 Gag CA [73]. The entire MHR region forms a compacted strand–turn–helix stabilized by a network of salt bridges and hydrogen bonds [74]. While this region (^284^DIRQGPKEPFRDYVDRFYKTL^304^) is not clearly understood [4,75], its presence has been suggested to have a role in CTD dimerization [23], thus contributing to the stability of the viral shell [76]. Although the MHR does not form part of the dimerization interface, it incorporates the binding region for some compounds that bind at this site [53]. It may therefore serve to interact with a cellular factor or viral component [74]. However, a structural study conducted on the SCAN domain (a dimerization element found in mammalian zinc-finger proteins with evolutionary and structural similarity to retroviral CTD of CA) suggests that the MHR contributes to the intertwined dimer interface via domain swapping [76]. The exchange of identical protein domains has been suggested as a mechanism for the evolution of oligomer interfaces as well as protein refolding and synthesis [77]. Ivanov et al. [78] showed that dimerization of a CA-swapped domain was perturbed via an alanine deletion. Mutations in this region may therefore affect viral assembly of the immature virus particles. Furthermore, the authors also suggest that a network of conserved hydrogen bonds may be implicated in the structural stability of the MHR and prevent protein unfolding [78]. In contrast, thermodynamic and kinetic experiments of domain-swapped CTDs by Bocanegra et al. [79] demonstrated that no correlation was observed between the MHR residues and protein domain swapping. Instead, the authors conclude that the MHR residues were alternatively imperative for stable CTD folding and therefore have important roles in immature CA assembly as well as other infectivity processes [79]. Though much research has been conducted on this region, it is evident that the true purpose of the MHR region is not yet known.

To conclude, as CA is involved in several stages of the viral replication cycle it is an attractive target for the development of inhibitory drugs. Consequently, therapeutic agents, such as Lenacapavir and PF74, have been shown to exhibit substantial anti-viral activity in both clinical [80] and in silico [81] studies, respectively.

## 4. Nucleocapsid

The nucleocapsid (NC; p7) protein, consisting of 55 amino acids [82], packages two copies of the viral RNA into rapidly assembling virions [83]. A prominent feature of NC is the presence of two highly conserved Cys-X2-Cys-X4-His-X4-Cys (CCHC) signatures that resemble zinc-finger motifs [83]. The zinc fingers, each comprising an aromatic residue (F16 in the N-terminal zinc finger and W37 in the C-terminal zinc finger) [84], coordinate a zinc ion [85] and are divided by a ^406^RAPRKKG^412^ basic domain linker [4]. These separately folded zinc fingers resemble beads attached to a string [22,86]. Nuclear magnetic resonance (NMR) studies indicate that the zinc fingers, positioned at the globular centre, are non-interacting and independently folded domains [86], whereas the N- and C-terminals of NC comprise a disordered structural conformation [82]. The chelation of zinc ions to the zinc fingers folds the central domain whilst the disordered N- and C-termini remain unfolded [87]. The flexible RAPRKKG linker between the W37 and F16 residues in NC modulates the spatial proximity of the globular structure [88]. In addition to these amino acids, residues V13, I24 and A25 in the first zinc finger and Q45 and M46 in the second zinc finger form a hydrophobic plateau in the central globular domain [87]. The hydrophobic plateau is essential for the interaction of small oligonucleotides and NC. Oligonucleotide–NC structural complexes show that the hydrophobic plateau allows NC to bind nucleic acids via multiple phosphate backbone and nucleotide base contacts [15]. In addition, W37 is stringently stacked with guanine, contributing to nucleic acid–NC binding energy [15]. Moreover, complex stability is further maintained through electrostatic interactions with the base amino acids in the zinc finger linker and the disordered NTD of NC [89]. Mutations affecting the ordered zinc finger folding and the formation of the hydrophobic plateau [85] modify the structure of the virion core, alter Gag trafficking [82] and result in the loss of viral infectivity [90]. For example, the substitution of P408 for D408 in the basic linker resulted in non-infectious, immature virions [91]. Although spatial closeness and orientation of the zinc fingers may be essential for successful virion production, only transient inter-zinc finger interactions were observed. Weak signal perturbations at the knuckle–knuckle (one turn α-helix followed by a β-hairpin) [92] interface of the zinc fingers were not consistent with tightly packed globular proteins [84,87,91,93].

### 4.1. NC and Genome Packaging

Genome packaging in HIV-1 is dependent on interactions between an un-cleaved precursor PR55 NC domain and a 110 nucleotide Psi (ψ; RNA packaging signal) segment [94] in the 5′ RNA leader sequence [83]. The ψ segment comprises four stem loops (SL1–SL4) that are connected by short linkers [95]. The stem loops are proposed to have independent but redundant functions [94] which include the facilitation of genomic RNA dimerization by the dimer initiation site (DIS) in SL1; the packaging of un-spliced mRNAs when the signal is overlapped with the ψ-site by the major splice donor (SD) in SL2; heterologous RNA packaging by SL3; and the adoption of alternate structures by SL4 [22]. Lu et al. [96] depicted SL2 and SL3 of the RNA packaging signal bound to NC. The second and fourth G residues in the SL3 tetra loop (677GGAG680) interact with both zinc fingers. The flexibility of the zinc finger linker allows the NC domain to interface with double or single stranded genomes [22]. Although both zinc fingers share similar conformation as well as hydrophobic and polar amino acid distributions, the two have completely different functions. The first zinc finger is essential for RNA packaging. The substitution of an amino acid sequence corresponding to the first zinc finger results in a 15% reduction of RNA packaging efficiency. Contrastingly, the second zinc finger has been highly associated with virion stabilization rather than encapsidation [89].

Although SL3 is important in RNA packaging, SL3-deficent ψ-sites only reduce the efficacy of genome packaging without complete elimination [97]. Similarly, mutations in SL3 and other SL ψ-sites do not result in a total loss of genomic packaging but reduce its ability to behave in this way [98].

Furthermore, it has been demonstrated that the nucleic binding and chaperone activity (NAC) associated with NC promotes thermodynamically stable conformations of RNA and DNA [96]. Consequently, NAC activity plays an important role in the conversion of genomic RNA (gRNA) to viral double-stranded DNA (dsDNA) [84]. Recently, Jiang et al. [82] showed that NC plays an integral role in the compaction of viral dsDNA within mature HIV-1 CAs, suggesting a pathway for de-condensation upon un-coating and NC loss.

### 4.2. Other NC Functions

Other functions of NC include the ability to renature nucleic acids with catalytic rates of about four orders of magnitude [99]. In reverse transcription, NC can also stimulate tRNA^Lys^ binding to the primer binding site found at the N-terminal region of the genome, initiate reverse transcription from the bound tRNA^Lys^ and partake in strand transfer [100]. Additionally, NC was shown to modulate RNA G-quadruplex stability through binding and unfolding of the RNA G-quadruplex as well as the promotion of DNA–RNA duplex formation, thereby allowing reverse transcription to proceed successfully [101].

## 5. p6

The C-terminal region of Gag comprises a proline-rich, 52 amino acid p6 domain [102] where two amino acid sequences are translated: the -1 frameshift Gag–Pol p6 and the in-frame Gag p6 domain [4]. Although p6 is largely considered to have a random structure in aqueous solution displaying little, if any secondary structure [67], Solbak et al. [103] showed that in a 100 mM dodecylphosphocholine micelle solution at pH 7, p6 forms defined N- and C-terminal helices that are connected by a flexible hinge. In spite of its debatable structure, it is widely accepted that p6 serves as a flexible docking site for cellular host factors [102,103,104]. HIV-1 p6 comprises two unique late domains [102]. As part of the precursor PR55 Gag polyprotein, these domains recruit and bind the endosomal sorting complex required for transport (ESCRT) cellular factor to promote the abscission of immature virions from the host cell [95]. The first late domain, YPXL (Tyr-Pro-X-Leu), binds cellular factor ALG2-interacting protein X (ALIX) [105]. Although the YPXL late domain varies between retroviruses, the conserved tyrosine always binds within a pocket on the ALIX V domain, and hydrophobic residues contact ALIX along a shallow groove [106]. Apart from p6, ALIX and its isolated Bro domain also interact with NC [107]. While this interaction could be RNA dependent, it may be non-specific [4]. The second late domain, PTAP binds the tumour susceptibility gene 101 (TSG101) cellular protein [67]. The four PTAP residues make contact with the N-terminal ubiquitin E2 variant (UEV) domain of TSG101 [108]. Not only are PTAP motifs found in related proteins that recruit ESCRT but also in the human HRS protein. Therefore, p6 can be regarded as a viral mimic of the cellular ESCRT-1 recruiting motif in HIV-1 [109].

Furthermore, while it is known that the precursor PR55 Gag NC domain mediates the selection of gRNA by interacting with the beginning of the *gag* gene gRNA and the ψ segment, the p6 domain also contributes to the RNA binding specificity of PR55 [110]. Using a truncated form of PR55 lacking the p6 domain, Dubois et al. [110] demonstrated a significant reduction in affinity for gRNA and high affinity for spliced cellular and viral RNAs. This suggests that the p6 domain has an essential role in the discrimination of viral and cellular RNAs from the selective gRNA required for encapsidation during immature particle assembly.

Interestingly, Yu et al. [111] demonstrated that point mutations at highly conserved residues, specifically at position F8 in the transframe p6 region of Gag–Pol, result in insufficient viral processing due to impaired protease and reverse transcriptase activity.

### 5.1. p6 and Vpr

During the late stage of replication, the p6 domain, as part of the un-cleaved precursor PR55 Gag, is also necessary for the incorporation of viral accessory protein, Vpr, into the virions [112]. The 96 amino acid protein is comprised of three amphipathic α-helices specifically arranged to form a hydrophobic centre surrounded by flexible sequences [113]. Propagation in HeLa cells showed that Vpr oligomer formation and its targeting to the nuclear envelope is mediated by this hydrophobic centre [114]. Vpr is associated with trans-activation of long terminal repeats (LTR), import of the PIC in non-dividing cells, the induction of apoptosis (programmed cell death) and cellular halt at the G_2_/M transition pathway [115]. The G_2_/M transition is a point in a cell’s lifecycle where, after the second growth phase (G2) and DNA replication (S phase), mitosis occurs (M phase) to separate the cell into identical daughter cells. In addition to translation being repressed, many of the cell’s components undergo a dramatic structural change that occurs during the G2/M transition pathway. This is imperative if identical daughter cells are to be produced [116]. Vpr is therefore critical in viral pathogenesis and must be incorporated in viral particles [117]. The ^41^LXXLF^45^ motif in p6 has been frequently associated with Vpr binding [102]. In contrast, the ^15^FRFG^18^ and ^34^ELY^36^ p6 motifs were found to be associated with Vpr packaging [104]. Wanaguru and Bishop [104] further demonstrated that removal of either the FRFG or LXXLF binding motifs coupled with the disruption of Vpr oligomerization significantly reduced Vpr incorporation by approximately 25- to 50-fold. Additionally, the authors also observed that although Vpr is concomitantly lost with p6 during infection, remnants of Vpr remain associated with CA for several hours, implicating Vpr functionality in early replication.

### 5.2. p6 and Phosphorylation

The p6 domain is also regarded as the primary phosphoprotein in HIV-1 and serves as a substrate for virus associated kinases, such as the extracellular signal regulated kinase 2 (Erk-2) targeting threonine; the atypical protein kinase C (aPKC) targeting serine [102]; and Elk tyrosine kinases [118]. The highly conserved S40 residue associated with phosphorylation has been related to core formation and capsid manipulation in infectious particles. However, the functional relevance of phosphorylation in p6 residues is still debatable [103]. Radestock et al. [119] attempted to elucidate the role of p6 phosphorylation by using a comprehensive mutational analysis. The analysis included a wild type proviral genetically uncoupled (unc) *gag* and *pol* plasmid (pNL4-3_unc_); a derivate with non-phosphorylation residues but with similar chemical structures (pNL4-3_unc_FL); and a derivative with wild type S40 and all other substitutions (pNL4-3_unc_FL-N40S). Controls included a derivate with a substituted alanine in FRFG and diminished Vpr incorporation; pNL4-3 Vpr(-), a derivate with a defective late domain; and pNL4-3 late(-) and a derivate that does not express Vpr (pNL4-3ΔVpr). The virus (NL4-3_unc_FL, NL4-3_unc_FL-N40S and S40N) displayed no significant change in replication capacity in comparison to the wild type virus (NL4-3_unc_). Consequently, the authors suggested that phosphorylation of p6 residues may not be essential for viral replication and morphogenesis, but may be structurally important as the small, hydrophilic serine is favoured over the hydrophobic, bulky phenylalanine [119]. Other authors have alternatively shown that the S40F substitution may diminish cleavage at the CA|SP1 site [120,121].

## 6. SP1 and SP2

The 14 amino acid SP1 (^364^AEAMSQVTNSATIM^377^), known interchangeably as p2, is found wedged between the N-terminal CA and C-terminal NC proteins [4]. The C- and N-termini of the CA and SP1 domains, respectively, were shown to form two parts of an α-helix [122]. Mutations in residues M367 and A366 within SP1 and K359 and H358 just outside SP1 were depicted to alter the conformation of the α-helix, subsequently inhibiting infectivity [123].

Although the structure of SP1 has not been resolved independently of the other Gag domains [67], current molecular studies show that SP1 has a propensity to form an α-helix with residues beginning from L343 in CA to V390 in SP1 directly linked to helix formation [14,124]. Interestingly, a circular dichroism analysis revealed that this helical conformation was considerably enhanced when 30% trifluoroethanol, a helix-promoting solvent, was subsequently added to the peptide solution after it had been purified. This conformational switch may imply the active role of SP1 in viral assembly [124]. However, as the primary assembly unit of the Gag lattice remains largely unknown [125], maintenance of this helical conformation in full length Gag is debatable. Electron cryotomography analyses were consistent with SP1 forming a six α-helical bundle that stabilized Gag hexamers in immature virus particles [126]. As a result, cleavage of SP1 from NC is necessary for the formation of ribonucleoprotein within RNA and condensation of the CA domain [127]. The late cleavage of CA|SP1 allows morphogenesis through CA–CA interactions. The importance of the integrity of the CA|SP1 segment in formation of immature CA was shown in an electron microscopic study [128].

In addition, the SP1|NC and CA|SP1 regions have been associated with Gag–Gag interactions as deletion of the peptide can lead to arrested ribonucleoprotein budding [123]. Atomic-level resolution structural studies on the CA|SP1 cleavage segment of Gag are necessary since this region not only contributes to virion assembly but may also have an important role in the HIV-1 maturation inhibitor binding site [129]. At present, inhibitors such as Bevirimat and PF-96 have been developed to block CA|SP1 cleavage during maturation [122]. However, natural polymorphisms, predominantly the V370A substitution in SP1, produce high level drug resistance to these inhibitors [130,131].

The 16 amino acid SP2 (^433^FLGKIWPSYKGRPGNF^448^), also known interchangeably as p1, lies between the CTD of NC and the NTD of p6. This peptide contains two highly conserved proline residues, namely P445 and P439 [4]. Hill et al. [132] implicated the importance of SP2 for Pol and Gag incorporation into immature virus particles. The “slippery site” involved in the Gag–Pol ribosomal frameshift overlaps with the end of SP2 leading to potentially complex effects of mutations at this site [4]. The substitution of either proline (P445 and P439) by leucines results in lower stability of the NC–RNA complex and rescinds infectivity [133]. Moreover, mutations at the cleavage sites at either end of SP2 have discrepant effects. For example, mutations in the p9 peptide (NC|SP2) have no effect on proviral integration, whereas mutations at the C-terminal that produces the p15 (NC|SP2|p6) or p8 (SP2|p6) peptides decrease the amount of integrated provirus on subsequent infection. This differential effect may suggest the role of p9 in proviral integration as opposed to p15 and p8 [134].

## 7. Conclusions and Future Perspectives

The Gag polyprotein and its individual subunits have significant roles in the lifecycle and perseverance of HIV-1. While full length Gag structures have not yet been resolved, which is partly due to their large molecular size, increasing our understanding of Gag at the structural level will further our knowledge on the elucidation of pathways to resistance and ultimately advancements in drug therapy. The complex, yet intricate roles that the Gag polyprotein and its individual subunits play in viral replication and infectivity, as reviewed in this article, indicate that while our understanding is not lacking, it is also not yet complete. Therefore, the following important aspects are still far from clear and requires extensive research:

1. As MA plays a crucial role in membrane targeting, as well as Env incorporation, it is a constantly evolving area of research. Extensive mapping of these independent yet linked pathways, especially where myristoylation is concerned, can possibly aid in the development of drug inhibitors targeting this stage in the viral lifecycle.
2. As CA proves to be one of the most dynamic Gag domains, it is imperative that the role of cellular host factors, such as CypA as well as the MHR region is understood to further research in the area of therapeutic agents. In addition, HIV-1 subtype-associated discrepancies in the role of CypA must be further explored.
3. The NC domain is an important component for nucleic acid packaging in immature viral particles. Its role in promoting stable nucleic acid conformations in the conversion–condensation–decondensation process requires extensive research to understand this mechanistic approach to replication.
4. p6 serves several functional roles in the overall viral replication process. One of the most intriguing aspects of p6 is its highly conserved residues and their role in interacting with viral and host cellular factors. Further understanding of these residues and their affinity for being phosphorylated can aid in the development of therapeutic targets for this conserved site.
5. The spacer peptides (SP1 and SP2) remain elusive. Though some important work has been conducted on these short segments, their role in viral replication and the maintenance of structural integrity is lacking. As with the main Gag domains, complex interactions are constantly being observed. However, what is evident is that the spacer peptides may not only serve supportive roles to the Gag domains, but may also have independent roles in viral assembly and infectivity on their own.

## Figures and Tables

**Table 1:** Summary of known functional roles employed by the HIV-1 Gag domains in viral replication and infectivity.

Gag Domain	Summarized Functional Roles
**Matrix;** **p17**	Facilitates targeting of Gag–Pol and Gag to the plasma membrane Incorporation of Env into virions Associated with reverse transcription and the pre-integration complex during early stages of replication Binds tRNA (transfer RNA), rRNA (ribosomal RNA) and heterologous DNA Acts as a viral cytokine to promote a favourable network of virus- and host-derived stimulatory factors for optimal infection and replication
**Capsid;** **p24**	Houses viral RNA and core-associated proteins Interaction with several cellular host factors and/or metabolites including cyclophilin A and inositol hexaphosphate (IP6) Interacts with cellular nucleoporins to allow import of the PIC The precursor PR55 Gag capsid domain and the mature p24 capsid display several functional roles in the viral replication cycle, including assembly, reverse transcription, integration and infectivity
**Nucleocapsid;** **p7**	Packages viral RNA into rapidly assembling, immature viral particles Ability to renature nucleic acids Stimulates tRNALys binding to the primer binding site, initiates reverse transcription from the bound tRNALys and partakes in strand transfer Possesses nucleic binding and chaperone activity in the conversion of gRNA (genomic RNA) to viral dsDNA (double-stranded DNA)
**p6**	Flexible docking site for cellular host factors, specifically ALIX and TSG101 Contributes to RNA binding specificity of gRNA of Gag PR55 Incorporation of viral accessory protein, Vpr Primary phosphoprotein Substrate for virus-associated kinases such as Erk-2 and Elk tyrosine kinases Contributes to RNA binding specificity of PR55 Gag
**SP1**	May have active roles in viral assembly and possibly infectivity Associated with Gag–Gag interactions in budding of immature virus particles
**SP2**	Plays a role in the incorporation of Pol and Gag in the immature virus particles Effect on the stability of nucleocapsid-RNA (NC–RNA) complexes and thus viral infectivity May have an effect on proviral integration when present as the p9 NC|SP2 peptide

## Data Availability

Not applicable.
